# TU-100 (Daikenchuto) and Ginger Ameliorate Anti-CD3 Antibody Induced T Cell-Mediated Murine Enteritis: Microbe-Independent Effects Involving Akt and NF-κB Suppression

**DOI:** 10.1371/journal.pone.0097456

**Published:** 2014-05-23

**Authors:** Nobuhiro Ueno, Takumu Hasebe, Atsushi Kaneko, Masahiro Yamamoto, Mikihiro Fujiya, Yutaka Kohgo, Toru Kono, Chong-Zhi Wang, Chun-Su Yuan, Marc Bissonnette, Eugene B. Chang, Mark W. Musch

**Affiliations:** 1 Department of Medicine, Knapp Center for Biomedical Discovery, University of Chicago, Chicago, Illinois, United States of America; 2 Division of Gastroenterology and Hematology/Oncology, Department of Medicine, Asahikawa Medical University, Asahikawa, Hokkaido, Japan; 3 Tsumura Research Laboratories, Tsumura and Co., Ami, Ibaraki, Japan; 4 Center for Clinical and Biomedical Research, Sapporo Higashi Tokushukai Hospital, Sapporo, Hokkaido, Japan; 5 Division of Gastroenterologic and General Surgery, Department of Surgery, Asahikawa Medical University, Asahikawa, Hokkaido, Japan; 6 Tang Center for Herbal Medicine Research, Department of Anesthesia and Critical Care, University of Chicago, Chicago, Illinois, United States of America; University of Chicago, United States of America

## Abstract

The Japanese traditional medicine daikenchuto (TU-100) has anti-inflammatory activities, but the mechanisms remain incompletely understood. TU-100 includes ginger, ginseng, and Japanese pepper, each component possessing bioactive properties. The effects of TU-100 and individual components were investigated in a model of intestinal T lymphocyte activation using anti-CD3 antibody. To determine contribution of intestinal bacteria, specific pathogen free (SPF) and germ free (GF) mice were used. TU-100 or its components were delivered by diet or by gavage. Anti-CD3 antibody increased jejunal accumulation of fluid, increased TNFα, and induced intestinal epithelial apoptosis in both SPF and GF mice, which was blocked by either TU-100 or ginger, but not by ginseng or Japanese pepper. TU-100 and ginger also blocked anti-CD3-stimulated Akt and NF-κB activation. A co-culture system of colonic Caco2BBE and Jurkat-1 cells was used to examine T-lymphocyte/epithelial cells interactions. Jurkat-1 cells were stimulated with anti-CD3 to produce TNFα that activates epithelial cell NF-κB. TU-100 and ginger blocked anti-CD3 antibody activation of Akt in Jurkat cells, decreasing their TNFα production. Additionally, TU-100 and ginger alone blocked direct TNFα stimulation of Caco2BBE cells and decreased activation of caspase-3 and polyADP ribose. The present studies demonstrate a new anti-inflammatory action of TU-100 that is microbe-independent and due to its ginger component.

## Introduction

The Japanese traditional medicine (Kampo) daikenchuto (TU-100) has been established to have anti-inflammatory, prokinetic, and blood flow effects in the gastrointestinal tract in both animal models as well as humans [1]–[14]. TU-100 is an extract from a mixture of ginseng radix, processed ginger, and Japanese green pepper (30%, 50%, 20% by weight). All three plant extracts contribute a number of active phytochemicals. Ginger contains several gingerols and shogaols (6-, 8-, and 10- isomers) that have anti-inflammatory and blood flow effects and are believed to act by modulating mitogen activated protein kinase (MAPK), protein kinase B (Akt), and NF-κB activities [15]–[19]. Japanese pepper contains hydroxy-sanshools (alpha and beta) that alter intestinal blood flow, motility, and barrier function by inducing adrenomedullin and calcitonin gene related peptides [3], [7], [8]. These compounds have been shown to activate intestinal epithelial TRPA1 channels [11]. Ginseng contains diverse compounds including protopanadiols and protopanaxatriols that exert anti-inflammatory effects. These and other ginseng-containing compounds modulate cell growth and act as anti-cancer agents [20]–[23]. In addition to these effects of individual extract constituents, TU-100 has been shown to activate nicotinic acetylcholine receptors, contributing to its effects on motility [13].

TU-100 has been shown to decrease intestinal inflammation in models of experimental colitis, including the trinitrobenzene sulfonic acid-induced colitis in the mouse and the adoptive transfer model of CD4^+^ CD45RB^high^ cells in the SCID knockout mouse [7], [10]. The anti-inflammatory actions of TU-100 were proposed to be multifactorial. Induction of adrenomedullin and CGRPs by the ginger shogaols and Japanese pepper sanshools appear to play a role since neutralization of adrenomedullin decreases the anti-inflammatory effects of TU-100 in TNBS colitis [7], [10]. Activation of TRPA1 channels may contribute to this effect of TU-100. The TU-100-induced blood flow effect is blocked by a CGRP antagonist (inhibits both adrenomedullin (a CGRP family member) and CGRP) and also blocked by antibody to adrenomedullin. The effect of TU-100 directly on intestinal epithelial cells is mediated by TRPA1. TU-100 effects CGRP also, but appears to be mediated via activation of TRPV1 on intestinal sensory nerves. Gingerols, shogaols and hydoroxysanshools are TRPV1 agonists [24. 25]. It has not been determined whether adrenomedullin neutralization blocks the effect of TU-100's effect on CGRP. Different components of TU-100 affect adrenomedullin differentially. Ginger compounds, especially shogaols, strongly stimulate TRPA1-mediated adrenomedullin release in normal rats [11] while hydroxysanshools, from Japanese pepper, have a similar but weaker effect in normal rodents. In the ischemic intestine, the effect of hydroxysanshools is greater in the diseased (ischemic) portions of intestine [8] while shogaols are not as effective in the ischemic intestine.

To extend our understanding of TU-100's anti-inflammatory effects, we investigated the actions of TU-100 in a model of T-cell mediated inflammation. In contrast to the TNBS- and CD4^+^ CD45RB^high^ adoptive transfer models, activation of CD3^+^ T cells in mice with anti-CD3 monoclonal antibody results predominantly in small bowel inflammation [26]–[30]. This was originally observed in humans treated with an anti-CD3 antibody to suppress organ transplant rejection. These patients developed a systemic cytokine response [31], [32]. Intraperitoneal injection of anti-CD3 antibody in mice appears to selectively activate small intestinal CD3^+^ T-lymphocytes and cause rapid pooling of intestinal contents (an effect called “enteropooling”) within 1–3 hours. This is followed by apoptosis of villus epithelial cells within 1.5–3 hours and induction of crypt epithelial cell apoptosis within 24 hours [26], [28]. Anti-CD3 antibody also increases TNFα levels in the small intestinal mucosa, an effect that appears essential to the development of enteritis, as anti-CD3 antibody treatment does not increase enteropooling or cause diarrhea in the TNFα receptor knockout mouse [27].

The present studies show TU-100 pre-treatment blocks jejunal enteropooling stimulated by anti-CD3 antibody, villus shortening, and subsequent development of enterocyte apoptosis. TU-100 also inhibits the induction of TNFα by anti-CD3 antibody. Notably, enteritis induced by anti-CD3 antibody is comparable in germ-free (GF) mice and their specific pathogen free (SPF) counterparts. Treatment with either TU-100 or the ginger component block anti-CD3 antibody-induced enteritis in GF mice, indicating that their effects in this model are independent of gut microbes.

## Materials and Methods

### Mouse studies and ethic statement

All animal work was approved by the University of Chicago Institutional Animal Care and Use Committee (Institutional Animal Care and Use Committee protocol 72101). C57Bl6/J mice were bred in house for all studies. Either specific pathogen free mice (SPF) or germ free (GF) were used. Mice were from 8–14 weeks of age and both genders were used. Mice were sacrificed using CO_2_ followed by cervical dislocation as approved by the University of Chicago Institutional Animal Care and Use Committee. TU-100 was included in diet AIN-76A at 15 gm/kg and mice were fed this diet for 3 days prior to treatment with anti-CD3 antibody (monoclonal 145 2C11 obtained from Fitch Monoclonal Antibody Core, Cancer Research Center, University of Chicago). Three days plus gavage one hour prior to anti-CD3 antibody injection was selected as preliminary experiments demonstrated maximal inhibition of enteropooling within this time. Mice were injected with 200µg antibody and sacrificed after 3 or 24 hours (these times points were selected on the basis of previous reports that defined the optimal times for different enteritis-associated changes (e.g. enteropooling, villus and crypt cell apoptosis) [27], [28]. A laparotomy was performed and a ligature placed around the intestine at the ligament of Treitz, and a second ligature carefully placed 3–4 cm distal to the first. This segment was then removed and the weight and length determined. Sections were fixed in formalin for determination of apoptosis by TUNEL staining (In Situ Death Kit, Roche, Indianapolis, IN) or stained with hematoxylin and eosin for histological examination for villus height and crypt depth using NIH Image J software. The imaging station included an embedded scale to calibrate length in microns. At least 20 villi and crypts in each section and 3 sections from each mouse were analyzed to determine villus height and crypt depth. Histological measurements were performed by two authors (NU, MWM) who were blinded to the treatment conditions for a given mouse. From adjacent sections, RNA was extracted using Trizol reagent according to the manufacturer's directions (Invitrogen, Carlsbad, CA) and protein was extracted as previously described [24].

### Epithelial immune cell coculture experiments

Human colonic adenocarcinoma Caco2BBE cells were grown as monolayers on permeable supports. Caco2BBE cells were a gift of Dr. Mark Mooseker, Yale University [33]. Cells were allowed to grow for 7 days to mature and then treated overnight with human IFNγ (100 U/ml) to increase expression of TNFα receptors. Human Jurkat-1 cells were seeded on the serosal/bottom side of the permeable support. After one day, the human anti-CD3 antibody UCHT1 (BD Biosciences) was added (200 ng/ml) and Jurkat-1 and Caco2BBE cells were harvested separately. The Jurkat cells were pelleted from the medium by centrifugation and the media analyzed for secreted human TNFα. Jurkat and Caco2BBE cells were extracted and cell lysates analyzed for indicated proteins by Western blotting as described above.

### TU-100 components

TU-100 or the ginger, ginseng, and Japanese pepper components were obtained as powders from Tsumura & Co. (Ibaraki, Japan). These powders lacked the maltose addition used in TU-100. Where indicated, AIN-76A diets were supplemented with 1.5% (wt/wt) TU-100. To ensure that mice had consumed diakenchuto, serum levels of TU-100 components were measured by Tsumura & Co. by LC/MC/MS. Compound K was routinely measured as confirmation or diakenchuto consumption since compound K has long plasma life compared with other components that are excreted in the urine. For gavage, either TU-100 or individual components were resuspended in water to provide 100 mg/ml TU-100, 50 mg/ml ginger, 30 mg/ml ginseng, or 20 mg/ml Japanese pepper extract, providing 10 mg TU-100 or 5 mg ginger, 3 mg ginseng, or 2 mg Japanese pepper per mouse (weighed and between 19–22 grams and volume adjusted per mouse). Mice were gavaged each day for three days with 100 μl of the above solutions and then one hour before i.p. injection of anti-CD3 antibody. These amounts are comparable to amounts consumed daily by humans.

### Protein analysis

Standard procedures were used for Western blotting and ELISA analysis of TNFα (eBioscience, San Diego, CA).

### Statistical methods

Data were analyzed using Graph Pad Prism software (San Diego, CA) using analysis of variance with a Bonferroni correction or paired Student's T test when appropriate.

## Results

### TU-100 decreases small bowel fluid enteropooling induced by treatment with anti-CD3 monoclonal antibody

**Figure 1:**
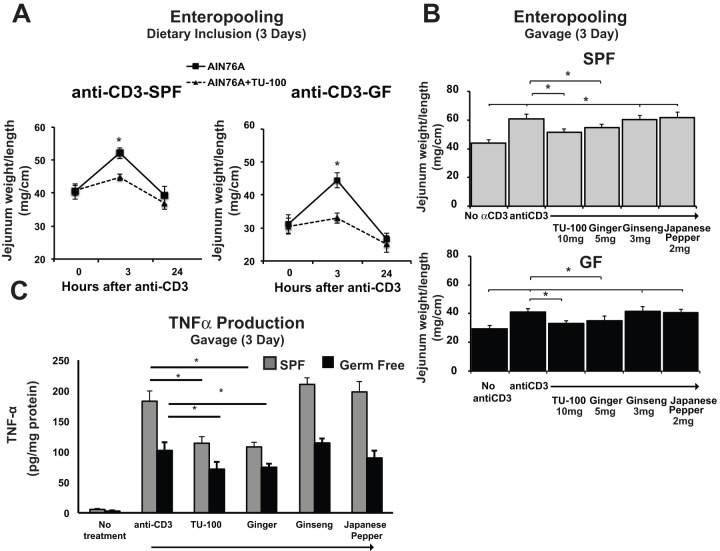
Dietary or gavaged TU-100 and the ginger component decrease enteropooling stimulated by anti-CD3 antibody treatment in specified pathogen free (SPF) and germ free (GF) C57Bl6 mice. Mice were fed AIN76A diet with or without TU-100 (A) or gavaged for three days plus one gavage one hour prior to anti-CD3 antibody with designated amounts of TU-100 or components (B and C). Three or twenty four hours after anti-CD3 antibody (dietary) or three hours after anti-CD3 (gavaged), length to weight measurements were taken in the proximal jejunum. Sections of tissue were homogenized and samples from the 3 hour time points used for TNFα determination by ELISA. Data are means ± SEM for 9 mice for dietary and 7 mice for each group in gavaged mice. For A, * p<0.05 by paired Student's t test. For B and C, * p<0.05, p<0.01 by analysis of variance using a Bonferroni correction.

Treatment with anti-CD3 antibody stimulated fluid accumulation within the jejunal lumen within 90–180 min. To determine if TU-100 prevented the enteropooling, mice were fed a diet with or without TU-100 (Figure 1A, 15 g TU-100/kg-chow) for 3 days. In separate experiments, mice were gavaged with TU-100 or the individual components of TU-100: ginger, ginseng, or Japanese pepper, to determine the effects of each component. Gavage was carried out daily for 3 days and then one hour prior to treatment with anti-CD3 antibody (Figure 1B). For specific pathogen-free mice (SPF contain normal intestinal microbiota) fed TU-100 diet (3 days), anti-CD3 antibody induced fluid enteropooling and distention of jejunal segments were significantly decreased (Figure 1A). To determine whether these changes were gut microbe-dependent, germ free (GF) C57Bl6 mice were fed sterile unsupplemented diet or diet supplemented with 1.5% TU-100 or gavaged with TU-100 or components that were autoclaved prior to gavage. As in SPF mice, anti-CD3 antibody treatment caused jejunal enteropooling that was blocked by dietary supplemented TU-100 (Figure 1A) or by gavage (Figure 1B). In both SPF and GF mice, ginger also blocked CD3 antibody induced enteropooling, but neither ginseng nor Japanese pepper were efficacious in this model (Figure 1B). As another measure of effects of TU-100 or TU-100 components in this model, we measured jejunal mucosal homogenate TNFα levels. Under basal conditions, TNFα levels were below ELISA assay detection limits, but TNFα levels increased three hours after anti-CD3 antibody treatment in both SPF and GF mice (Figure 1C). TU-100 or ginger gavage for three days blocked the stimulated expression of TNFα (Figure 1C). As in the case of enteropooling, ginseng and Japanese pepper components did not have any effects.

**Figure 2:**
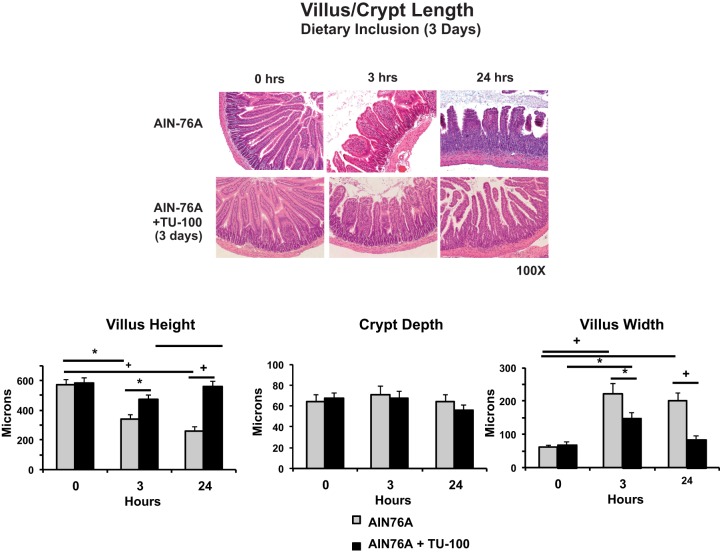
Dietary TU-100 blocks villus edema and shortening induced by anti-CD3 antibody. Data presented is from SPF mice fed AIN76A diet with or without TU-100 for 3 days, injected with anti-CD3 antibody and used for enteropooling assays of Figure 1. After weight and length measurements of the intact jejunal tissue, sections were formalin fixed for hematoxylin and eosin staining and villus and crypt dimensions measured using Image J software. Data are means ± SEM of 9 mice in each group. * p<0.05 by analysis of variance using a Bonferroni correction.

Anti-CD3 antibody treatment also injured the jejunal mucosa, with villus edema detectable within 3 hr and further increased by 24 hr and villous shortening was also present by this time point (Figure 2). Impressively, dietary TU-100 prevented the development of villus edema and shortening stimulated by anti-CD3 antibody treatment. There was little or no infiltration of neutrophils in this model at either 3 or 24 hr after anti-CD3 antibody treatment as assessed by histological examination or measurements of myeloperoxidase activity. No ulcerations were observed.

### TU-100 effects on mucosal apoptosis

#### TU-100 decreases apoptosis stimulated by anti-CD3 antibody treatment

**Figure 3:**
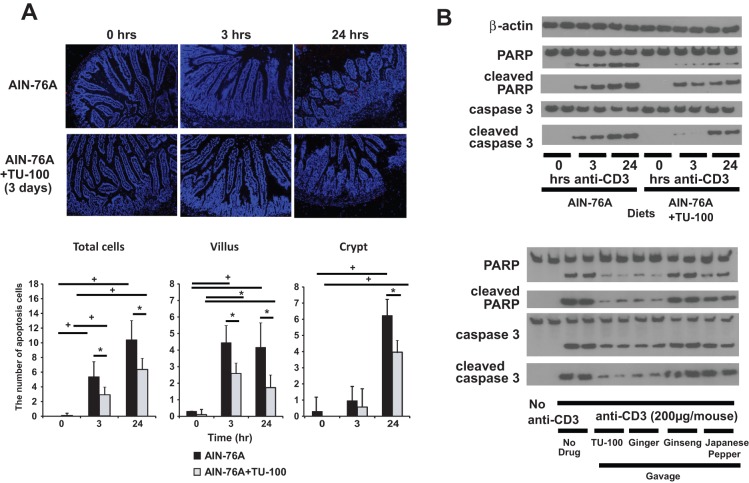
Dietary TU-100 decreases apoptosis induced by anti-CD3 antibody. A section of tissues from mice where enteropooling was determined were formalin fixed for H&E staining as well as TUNEL staining for apoptosis (A) and an adjacent section homogenized for Western blot analysis of apoptotic proteins (B). Apoptotic cells were counted per villus or crypt using Image J software. Images shown and means ± SEM are representative of 9 mice. For Western blotting (B) two mice from each group are presented and image is representative of 6 mice. * p<0.05+p<0.01 by analysis of variance using a Bonferroni correction.

Anti-CD3 antibody was shown to stimulate apoptosis of jejunal epithelial cells. Cell death first occurred in villous enterocytes by 3–8 hrs, followed by apoptosis of crypt enterocytes within 24 hrs [28]. In the current study we found that anti-CD3 antibody treatment increased apoptosis in jejunal villus epithelial cells within 3 hrs as assessed by TUNEL staining. Apoptotic injury was significantly reduced by dietary TU-100 (Figure 3A). Apoptotic crypt cells were observed at 24 hrs, and TU-100 decreased crypt cell death. TUNEL staining positive cells were quantified using NIH Image J software for quantification. Apoptosis was also assessed by the appearance of proteolytically cleaved forms of caspase-3 and polyADP ribose polymerase (PARP)(Figure 3B). Anti-CD3 antibody treatment induced cleavage of both caspase 3 and PARP, and these effects were reduced by dietary TU-100 (Figure 3B).

To determine the ability of TU-100 components to block anti-CD3 antibody-stimulated apoptosis, mice were gavaged daily and 1 hr before anti-CD3 antibody treatment with the indicated TU-100 components. As in the case of dietary TU-100, TU-100 gavage blocked cleavage of caspase 3 and PARP (Figure 3B). Among the components, ginger blocked the enteropooling and apoptosis induced by anti-CD3 antibody treatment to nearly the same extent as TU-100. Ginseng, in contrast, had no effect, and Japanese pepper extract exerted only a modest effect (Figure 3B).

#### TU-100 and ginger block anti-CD3 antibody activation of Akt

**Figure 4:**
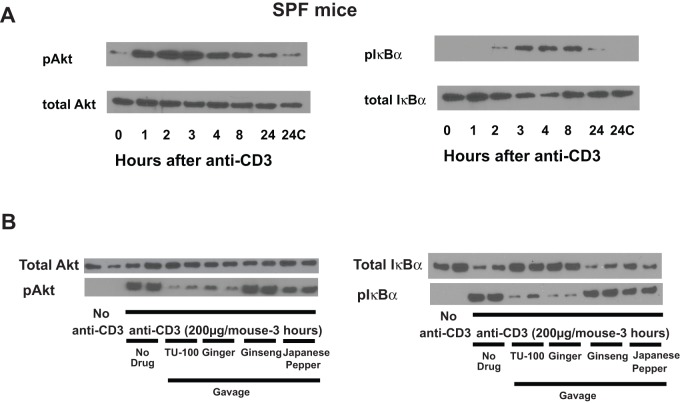
TU-100 blocks activation of jejunal mucosal Akt and IκB stimulated by anti-CD3. (A) Mice were sacrificed at varying times after anti-CD3 antibody injection. Blots are representative of three separate mice. (B) Mice were gavaged daily for 3 days and one hr before anti-CD3 antibody treatment with TU-100 or components. Mice were sacrificed 3 hrs after anti-CD3 antibody injection. Blots are representative of two mice for each group, and a total of 6 mice were analyzed.

In lymphocytes, anit-CD3 antibodies activate protein kinase B, also termed Akt. Because gingerols have been demonstrated to inhibit Akt, the effects of TU-100 and its components on anti-CD3 antibody-induced Akt activation were determined as assessed by its state of phosphorylation (Thr 308). The time course of Akt activation in jejunal homogenates was determined in intact jejunum following injection with anti-CD3 antibody. Akt activation was detectable within one hour and maximal by 2–3 hrs (Figure 4A). We therefore chose 3 hrs to study the effect of TU-100 and its constituent extracts on anti-CD3 antibody induced Akt activation. Mice were gavaged daily for 3 days and one hour prior to anti-CD3 antibody treatment. TU-100, ginger and, to a lesser degree Japanese pepper, blocked anti-CD3 antibody induced Akt activation. In contrast, ginseng had no effect (Figure 4B).

Gingerols and shogaols have also been reported to block NF-κB and Akt activation [34], [35]. Akt activation has been associated with anti-CD3 activation and NF-κB associated with TNFα stimulation [36], [37]. Anti-CD3 antibody treatment stimulated mucosal NF-κB activation as assessed by phosphorylation of NF-κB inhibitor IκBα (pIκBα) that was nearly maximal after 3–4 hours but decreased after 8 hrs (Figure 4A). Similar to effects on Akt, both TU-100 and ginger were equally effective in blocking the anti-CD3 antibody activation of NF-κB, whereas Japanese pepper showed a more modest inhibitory effect (Figure 4B).

#### Diakenchuto and ginger block anti-CD3 antibody induced activation of T-lymphocyte Akt and subsequent TNFα activation of epithelial NF-κB

**Figure 5:**
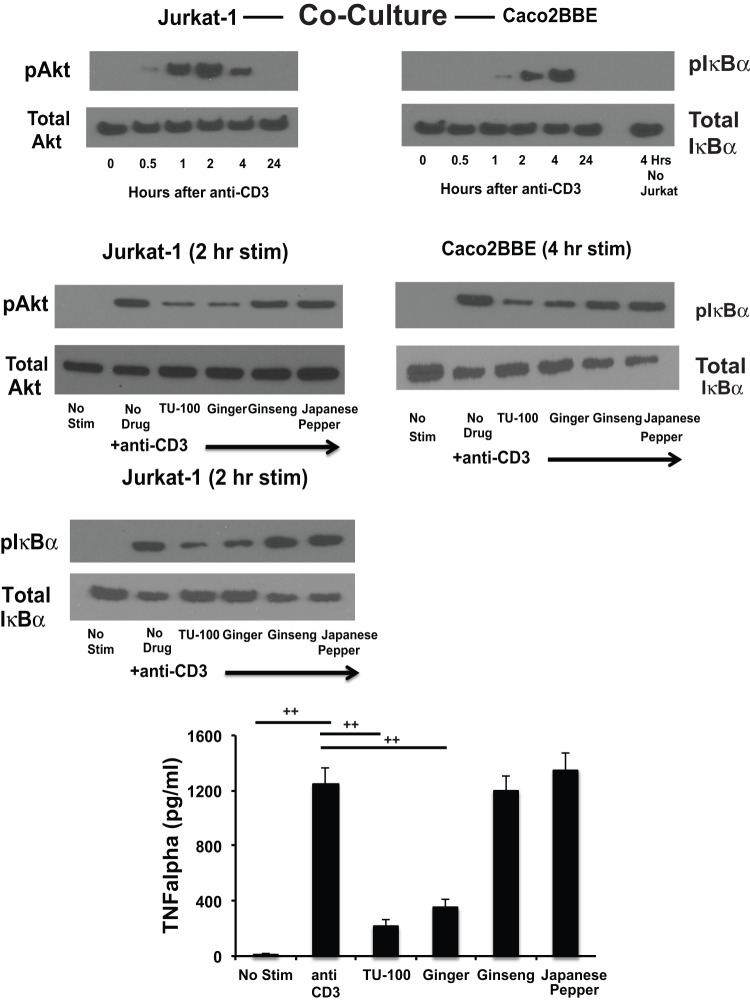
TU-100 blocks Akt and NF-κB stimulation by anti-CD3 antibody in Jurkat cells and NF-κB stimulation in Caco2BBE cells. Caco2BBE cells were grown on permeable supports until confluent, differentiated, and then placed over human Jurkat-1 T lymphoma cells that were stimulated with UCHT1 anti-human CD3 antibody. For one Caco2BBE well, only medium with UCHT1 antibody was used, no Jurkat-1 cells. Cells were harvested at indicated times after co-culture and proteins analyzed by Western blotting. For TNFα measurements, the Jurkat-1 conditioned medium was analyzed. Data are means ± SEM for 4 separate experiments. ++p<0.01 by analysis of variance using a Bonferroni correction.

To determine the effects of TU-100 and ginger on Akt and TNFα activation of T cells by anti-CD3 treatment, a co-culture system of human T-lymphocyte and human colonic epithelial cells was employed. Human colonic Caco2BBE cells were grown on a permeable support so that human T-lymphocytes (Jurkat-1) could be co-cultured with them. Caco2BBE were seeded and grown on polyethylene terphalate wells and allowed to mature for 7 days. Caco2BBE were then treated overnight with human IFNγ to increase TNFα receptor expression and response to TNFα. Jurkat-1 (5 million) cells were seeded in the lower compartment of the co-culture set up, i.e. without direct contact with the Caco2BBE cells in the upper compartment. After 24 hours, TU-100 or its separate components were added to the medium (both mucosal and serosal) and after 2 hours the anti-human CD3 antibody was added (200 ng per well). Jurkat-1 and Caco2BBE cells were then harvested and analyzed separately. The lower compartment medium was also recovered for TNFα measurement. Anti-human CD3 antibody activated Jurkat-1 cell Akt within 2 hours, an effect that could be inhibited by prior treatment with either TU-100 or ginger (Figure 5). Increases in T cell TNFα were also blocked by prior addition of either TU-100 or ginger (Figure 5). Stimulation of Caco2BBE NF-κB occurred in a time-dependent manner following Jurkat-1 treatments. The addition of anti-CD3 antibody to Caco2BBE cells in absence of Jurkat-1 cells did not activate NF-κB (Figure 5). Thus, CD3 antibody effects on intestinal epithelial cell NF-κ is T cell-dependent.

**Figure 6:**
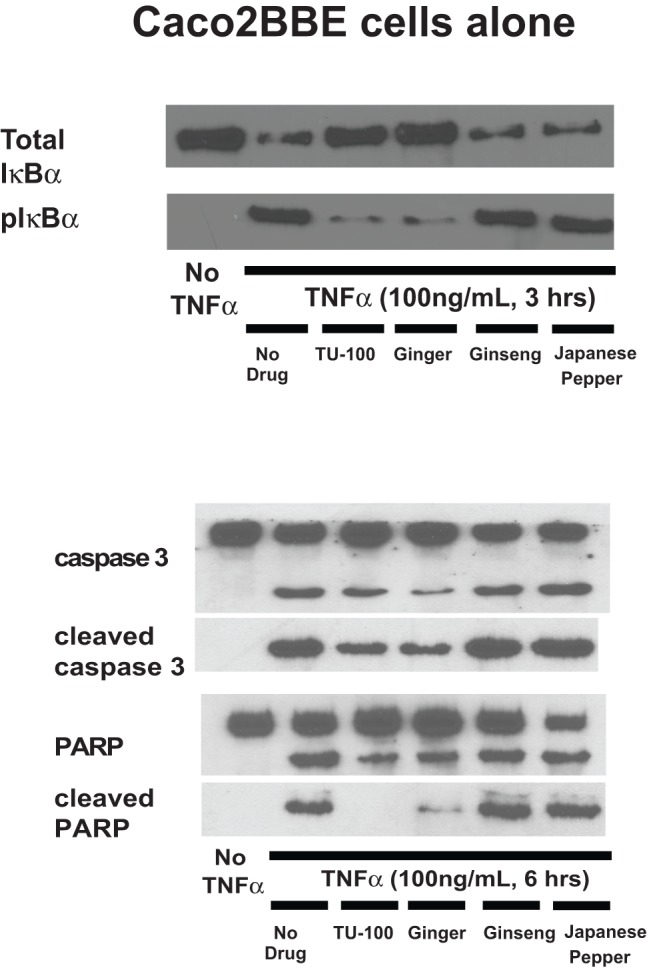
TU-100 blocks TNFα activation of IκBα, caspase 3 and PARP in Caco2BBE cells. Caco2BBE cells were treated overnight with IFNγ to increase TNF receptor expression and then stimulated with TNFα (100 ng/ml). Cells were harvested at 3 hrs for IκBα determinations and 6 hours for caspase 3 and PARP. Blots shown are representative of four separate experiments.

The T cell-mediated inhibition of the anti-CD3 antibody activation of Caco2BBE NF-κB by TU-100 could be due to reduced TNFα levels. To address this question, we stimulated Caco2BBE cells with a high concentration of TNFα. Caco2BBE were also treated with IFNγ to increase TNF receptor expression and with TU-100 or its components. Caco2BBE were then stimulated with TNFα (100 ng/ml) for 3 hrs (IκB activation) or 6 hrs (caspase 3 and PARP activation). Pretreatment of Caco2BBE cells with either TU-100 or ginger blocked the TNFα-mediated increases in phosphorylation of IκBα (Figure 6). As a high concentration of TNFα was used, significant decreases in total IκBα were consistently observed in the absence of TU-100. To determine whether the functional consequence of inhibited NF-κB activation, cells were incubated with TNFα for 6 hours to assess apoptotic activity that was measured by caspase 3 and PARP cleavage. These apoptotic proteins were activated following TNFα stimulation and TU-100 which was blocked by ginger (Figure 6).

## Discussion

Anti-CD3 antibody treatment induces a unique type of acute enteritis that is dependent on T cells and specifically appears to be regulated by lamina propria CD3^+^ CD4^+^ lymphocytes. The present study demonstrates for the first time that anti-CD3 antibody induced enteritis also occurs in germ free mice. Therefore this intestinal inflammation is microbe-independent, unlike other models of colitis such as CD45RB^hi^ cell adoptive transfer, piroxicam treatment in mice (only acute phase occurs in germ free mice), or the HLA B27 rat colitis. The Japanese traditional medicine (Kampo) TU-100 and one of its constituent components, ginger, inhibited enteropooling in both the SPF and GF mice. Therefore our studies demonstrate that gingerols or shogaols are the active agents in TU-100 that inhibit inflammation in this model. Additionally, as the effects were observed in germ free mice, the actions of these agents are also independent of intestinal bacteria.

Several signal transduction proteins activated in this model are blocked by TU-100 or ginger, including Akt and NF-κB. We demonstrate that anti-CD3 antibody activation of Akt and subsequent stimulated production of TNFα by CD4^+^ lamina propria lymphocytes are relevant in this model. Additionally, the enteropooling effect requires epithelial cell NF-κB activation [29], [30], therefore both the CD3^+^CD4^+^ T cells and intestinal epithelial cells are likely to affected by TU-100.

The studies demonstrate for the first time that anti-CD3 antibody induction of enteritis is independent of microbes. We also demonstrated that TU-100 or its constituent compound ginger exert therapeutic efficacy to block this enteritis. Among the diakenchuto components, the gingerols/shoagaols and sanshools are not known to require microbial metabolism for activity. Our study would also support this conclusion. Gingerols/shoagaols and sanshools are rapidly absorbed within 30 minutes after TU-100 ingestion, suggesting this occurs in the proximal gastrointestinal tract prior to exposure to the majority of the intestinal microbiome that resides in the colon [38], [39]. In contrast for ginseng, it is known that many ginseng compounds require bacterial metabolism, such as conversion of ginsenoside Rb1 to compound K, as well as other ginsenoside conversions. We and others have shown that compound K has potent anticancer effects mediated by the microbial metabolites of certain ginsenosides [21], [40]–[42].

This is only the second study to investigate the actions of TU-100 on small intestinal inflammation. A prior study had shown that small intestinal damage induced by the drug CPT-11 is also inhibited by TU-100 [43]. Whether TU-100 can be used to treat other small bowel inflammatory diseases such as viral enteritis or Celiac disease remains to be determined. Further studies are needed to determine the mechanism by which gingerols or shogaols inhibit Akt and NF-κB. It is possible that the effects of ginger as well as TU-100, may be due to their antioxidant activities [44]–[46] which could also inhibit NF-κB and Akt signals. These results demonstrate how the effects of complex substances such as TU-100 can be dissected to understand the contribution of individual components given appropriate model systems that respond to this agent. Such studies can be invaluable to extend our mechanistic understanding of these widely used complex combinations of phytochemicals.

## Supporting Information

Table S1 **Loop Summary.**
(XLS)

Table S2 **Tissue TNFα.**
(XLS)

Table S3 **Intestinal Morphometrics.**
(XLS)

Table S4 **Apoptosis TUNEL.**
(XLS)

Table S5 **TNFα C2JK.**
(XLS)
